# Unveiling Alterations of Epigenetic Modifications and Chromatin Architecture Leading to Lipid Metabolic Reprogramming during the Evolutionary Trastuzumab Adaptation of HER2‐Positive Breast Cancer

**DOI:** 10.1002/advs.202309424

**Published:** 2024-03-09

**Authors:** Ningjun Duan, Yijia Hua, Xueqi Yan, Yaozhou He, Tianyu Zeng, Jue Gong, Ziyi Fu, Wei Li, Yongmei Yin

**Affiliations:** ^1^ Department of oncology First affiliation hospital of Nanjing medical university Nanjing 210029 China

**Keywords:** 3D genome architecture, breast cancer, histone modification, metabolic reprogramming, secondary trastuzumab resistance

## Abstract

Secondary trastuzumab resistance represents an evolutionary adaptation of HER2‐positive breast cancer during anti‐HER2 treatment. Most current studies have tended to prioritize HER2 and its associated signaling pathways, often overlooking broader but seemingly less relevant cellular processes, along with their associated genetic and epigenetic mechanisms. Here, transcriptome data is not only characterized but also examined epigenomic and 3D genome architecture information in both trastuzumab‐sensitive and secondary‐resistant breast cancer cells. The findings reveal that the global metabolic reprogramming associated with trastuzumab resistance may stem from genome‐wide alterations in both histone modifications and chromatin structure. Specifically, the transcriptional activities of key genes involved in lipid metabolism appear to be regulated by variant promoter H3K27me3 and H3K4me3 modifications, as well as promoter‐enhancer interactions. These discoveries offer valuable insights into how cancer cells adapt to anti‐tumor drugs and have the potential to impact future diagnostic and treatment strategies.

## Graphical abstract

Secondary trastuzumab resistance signifies an evolutionary adaptation of HER2‐positive breast cancer during anti‐HER2 treatment. The reprogramming of cellular metabolic pathways accompanying this process are driven by global epigenetic alterations, including histone modifications on promoters and enhancers, as well as chromatin interactions between these regions.

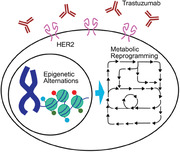

## 1 Introduction

Breast cancer is among the most prevalent malignancies affecting women worldwide, while the HER2‐positive subtype accounts for approximately 25% of all breast cancer cases.^[^ ^1^ ^]^ Currently, anti‐HER2 treatments are widely employed in the management of HER2‐positive breast cancer.^[^ ^2^, ^3^ ^]^ These treatments encompass not only traditional trastuzumab, which directly inhibits HER2 dimerization and downstream signal cascades, but also trastuzumab‐derived antibody‐drug conjugates (ADCs) to induce additional anti‐tumor cytotoxic effects.^[^ ^4^ ^]^

However, a significant proportion of patients either do not respond to trastuzumab, termed as “primary resistance,” or initially benefit from treatment but later lose these clinical benefits, referred to as ′secondary resistance’.^[^ ^5^, ^6^ ^]^ Both situations significantly diminish the effectiveness of trastuzumab and related ADCs through several mechanisms but the later one even worse. These mechanisms include HER2 mutations, abnormal activation of HER2 downstream or bypass signaling pathways, and immune suppression within the tumor microenvironment (TME).^[^ ^5^ ^]^ Yet, primary trastuzumab resistance is attributed to inherent properties of cancer cells, which mainly caused by mutation and gene rearrangement of HER2 and members of relevant signal cascades,^[^ ^7^, ^8^ ^]^ while secondary trastuzumab resistance results from adaptive changes that occur during long‐term treatment, which can be likened to an evolutionary process with selective pressure.

Throughout this evolutionary process, in addition to direct drug resistance, numerous indirect cellular processes also undergo changes,^[^ ^9^, ^10^, ^11^, ^12^ ^]^ which grant cancer cells increased flexibility within the tumor microenvironment (TME). Among these processes, metabolic reprogramming plays a pivotal role, enabling cancer cells to acquire metabolic properties that support their survival, immune evasion, and metastasis, therefore, to be a crucial area of research.^[^ ^13^, ^14^, ^15^ ^]^ For instance, changes in energy‐related pathways such as glycolysis and oxidative phosphorylation (OXPHOS) enable cancer cells to meet their ATP requirements in complex environments.^[^ ^16^, ^17^, ^18^, ^19^ ^]^ Additionally, alterations in carbohydrate, amino acid, lipid, and other nutritional metabolism pathways serve not only as sources of precursors for cellular component synthesis but also as suppliers of signal molecules that regulate the activities of both cancer cells and neighboring cells.^[^ ^20^, ^21^, ^22^, ^23^, ^24^, ^25^ ^]^ However, there is still much to learn about how breast cancer cells undergo metabolic adjustments during the development of trastuzumab resistance.

The adaptative evolution of cancer cells to anti‐tumor drugs can be influenced by a combination of genetic and epigenetic factors. Typically, mutations, especially somatic mutations and gene amplifications, are the most common drivers of breast cancer evolution.^[^ ^26^, ^27^, ^28^ ^]^ However, an increasing body of research has underscored the significance of epigenetic alterations in causing abnormal expression changes in both proto‐oncogenes and tumor suppressor genes.^[^ ^27^, ^29^, ^30^, ^31^, ^32^, ^33^ ^]^

Epigenetic alterations encompass modifications on genomic DNA especially CpG islands and histones as well as arrangements of DNA structure, nucleosome location and more complicated 3D genome architecture.^[^ ^34^, ^35^, ^36^, ^37^, ^38^ ^]^ These coordinated changes play a crucial role in regulating gene expression and constitute another significant force driving cancer evolution. Nevertheless, our understanding of how epigenetic alterations function during the adaptative evolution of HER2‐positive breast cancer cells to trastuzumab remains limited.

In this study, we aimed to uncover how breast cancer cells reorganize their cellular metabolism during the formation of secondary trastuzumab resistance. More importantly, we sought to understand the corresponding epigenetic alterations by conducting a comprehensive analysis, which included not only transcriptomic and epigenomic data but also 3D genome architecture information. We compared these data between original trastuzumab‐sensitive cells and secondary trastuzumab‐resistant cells. Our findings indicate that the properties of breast cancer cells and their interactions with neighboring cells can be influenced by global metabolic reprogramming, especially in relation to lipid metabolism. While alterations in histone modifications at promoter regions, as well as changes in promoter‐enhancer contacts that influenced by chromatin structure rearrangements, are pivotal factors driving these transformations.

## 2 Experimental Section

### 2.1 Cell Culture and Induction of Secondary Trastuzumab Resistance

Primary HER2‐positive trastuzumab‐sensitive breast cancer SKBR3 cells and trastuzumab‐resistant breast cancer JIMT1 cells were cultured in modified Eagle's medium (DMEM) (Gibco, 11960044) supplemented with 10% fetal bovine serum (FBS) (Gibco, 16140071), 80 U ml^−1^ penicillin, and 0.08 mg ml^−1^ streptomycin (Gibco, 15140122) at 37 ^◦^C in a humidified environment with 5% CO_2_.

Secondary trastuzumab‐resistant SKBR3_HR cells were induced from SKBR3 cells by raising the concentration of trastuzumab gently from 1 to 20 ug ml^−1^ in 30 weeks period to prevent acute inhibition of cell growth. The original SKBR3 cells and primary trastuzumab‐resistant JIMT1 cells were also cultured parallelly for 30 weeks as control. Each cell type holds 3 biological repeats.

### 2.2 Cell Viability Assay

Cell viability was performed by CCK‐8 assay. 5000 cells of each type were seeded in a 96‐well plate. After 24 h incubation at 37 ^◦^C in a humidified environment with 5% CO_2_, old medium was replaced by new medium containing gradient diluted trastuzumab. After another 48 h culture, new medium with CCK‐8 reagent (Vazyme, A311) was applied to replace the old medium. After 1 h incubation, the absorbance of each well at 450 nm wavelength was measured by a multi‐well plate reader.

### 2.3 Extracellular PGE2 Quantification

Extracellular PGE2 was measured by ELISA assay (Dogesce, DG10157H‐48). In brief, 2 ml fresh medium was used to replace the old medium in 6‐well plates planted with cells in advance and then collected after 48 h incubation. After centrifuging at 12 000 rpm for 10 min at 4 °C, 50 µl supernatant was transferred into an ELISA plate coated with anti‐human PGE2 antibody. After adding HRP‐conjugated anti‐human PGE2 antibody and 1 h incubation at 37 °C, the plate was washed with wash buffer 5 times. After adding of 50 µl TMB substrate to each well and another 10 min incubation at 37 °C, 50 µl stop solution was then added to each well. The absorbance of each well at 450 nm was measured by a multi‐well plate reader. The final PGE2 concentration was normalized by the total cell number in each well.

### 2.4 PBMC Isolation and Co‐Culture assay

Human peripheral blood mononuclear cells (PBMCs) were collected from blood of healthy donors by density gradient centrifugation with Ficoll medium (Sigma, F4357). Isolated PBMCs were stored in 90% FBS with 10% DMSO at −80 °C.

PBMCs were activated by 1:100 diluted T Cell TransAct (Miltenyi Biotec, 130‐111‐160) and 20 IU ml^−1^ human IL‐2 (Sigma, SRP3085). Activated PBMCs were cultured in RPMI 1640 medium (Gibco, 11875101) supplied with 5% FBS, 80 U ml^−1^ penicillin and 0.08 mg ml^−1^ streptomycin at 37 °C with 5% CO_2_.

SKBR3 and SKBR3_HR cells were seeded in 96‐well plate with DMEM for 24 h before co‐culture with PBMCs. Activated PBMCs were added to each well at a ratio of 20:1 with different trastuzumab concentrations, and PGE2 was added to the medium of SKBR3 cells to reach the same concentration as SKBR3_HR cells. After another 48 h incubation, PBMCs were removed, and the viability of cancer cells were evaluated by CCK‐8 assay.

### 2.5 Western Blots

Cells were washed with PBS after collection and lysed in SDS lysis solution supplied with protein inhibitor cocktail (Beyotime, P0013G) on ice for 5 min. Lysates were centrifuged at 12 000 rpm for 10 min at 4 °C. The supernatants were mixed with 5X loading buffer (Beyotime, P0015) and boiled at 95 °C for 15 min. Proteins were separated by SDS‐PAGE with 15% percentage gels, transferred to PVDF membranes (Bio‐Rad, 1620177) and blocked in 5% skim milk in TBST (20 mm Tris, 150 mm NaCl, and 0.1% Tween‐20) for 1 h. Membranes were incubated in TBST with primary antibodies (H3K27me3(CST, C36B11), H3K4me3(Abcam, ab213224), H3K27Ac (Abcam, ab177178) and Histone H3(Abcam, ab1791)) at 4 °C overnight. After washed with TBST 3 times, membranes were incubated in TBST with horseradish peroxidase (HRP) conjugated anti‐rabbit secondary antibody at room temperature for 1 h. After being washed with TBST 3 times, protein bands were visualized in Western Blot imaging system with ECL chemiluminescence kit (Vazyme, E422‐01) and quantified using ImageJ.

### 2.6 Sequencing Library Preparation for CUT and Tag Assay

Genomic histone modification measurement was performed by cleavage under targets and tagmentation (CUT & Tag) sequencing with a commercial CUT & Tag assay kit (Vazyme, TD903). In brief, 10^5^ cells with >90% viability were obtained for each test. After binding with magnetic Concanavalin A beads, cells were permeabilized and treated with primary antibodies (H3K27me3(CST, C36B11), H3K4me3(Abcam, ab213224), H3K27Ac (Abcam, ab177178), Rad21 (Abcam, ab217678) and CTCF (CST, 3418T)) and the following goat anti‐rabbit secondary antibody separately. After that, genome DNA of each sample was first digested by protein A/G bonded Tnp, then released by proteinase K treatment and purified with SPRIselect beads (Vazyme, N411). Spike‐in DNA was added according to the input cell number (1 ng per 100 000 cells) for coverage normalization between different samples. Library amplification was performed by an Illumina‐compatible index kit (Vazyme, TD202), and the following sequencing was carried out by an Illumina Novaseq 6000 platform.

### 2.7 Sequencing Library Preparation for Micro‐C Assay

The Micro‐C library was prepared using the Micro‐C Kit (Dovetail #21006) according to the manufacturer's protocol. Briefly, the chromatin was fixed with disuccinimidyl glutarate (DSG) and formaldehyde in the nucleus. The cross‐linked chromatin was then digested in situ with micrococcal nuclease (MNase). Following digestion, the cells were lysed with SDS to extract the chromatin fragments and the chromatin fragments were bound to Chromatin Capture Beads. Next, the chromatin ends were repaired and ligated to a biotinylated bridge adapter followed by proximity ligation of adapter‐containing ends. After proximity ligation, the crosslinks were reversed, the associated proteins were degraded, and the DNA was purified then converted into a sequencing library using Illumina‐compatible adaptors. Biotin‐containing fragments were isolated using streptavidin beads prior to PCR amplification. The library was also sequenced on an Illumina Novaseq 6000 platform.

### 2.8 Bioinformatics Analyses

#### 2.8.1 RNA‐Seq Data Analyses

RNA‐seq reads were aligned to the human hg38 reference genome. Differential expression analyses were performed by the DEseq2 package in R on the count data of each cell type. The threshold of significantly differential expression was defined as Padj < = 0.05 & |log2 fold change| > = 0.5.

### 2.9 Metabolism Pathway Activity Calculation

The activity score of metabolism pathways among different cell types was calculated according to Xiao's work.^[^ ^39^ ^]^ In brief, the mean expression level of each metabolic gene across each cell type (contains 3 biological repeats) was calculated first, while its relative expression level in each cell type was defined as the ratio of its mean expression level to its average across all cell types, and the activity score of a certain pathway in a certain cell type was calculated as the weighted average of relative gene expression level over all genes in this pathway.

### 2.10 CUT and Tag Analysis

CUT and Tag reads were aligned to the human hg38 reference genome with Bowtie2 (v2.2.5) and then filtered and converted to.bed files by Samtools (v1.14) and Bedtools (v2.30.0). SEACR (v1.3) was applied for peak calling while chromVAR together with DEseq2 packages in R was used for differential analysis. Significantly changed peaks were defined as Padj < = 0.05 & |log2 fold change| > = 0.5.

### 2.11 Micro‐C Data Analysis

#### 2.11.1 Micro‐C Data Process

Raw paired sequencing reads of Micro‐C were aligned to the human hg38 reference genome assembly with BWA‐MEM (v0.7.17). The following ligation junction finding, sorting and PCR duplicates removal were all performed with pairtools (v1.0.2). Juicertools (v1.22.1) and cooler (v0.9.2) were applied to generate.hic and.mcool contact matrices. Stratum‐adjusted correlation coefficient (SCC) indicating the concordance between biological replications of each cell type was calculated by the hicrep package in R.^[^ ^40^ ^]^ The P‐s curve was calculated using cooltools (v0.5.4) as a function of intra‐chromosome contact frequency (P) and genomic distance (s).

#### 2.11.2 TAD and Loop Analyses

Micro‐C contact matrices were used to identify TAD and loop with HOMER (v4.11). Relevant insulation scores were also provided within this step. We collected merged TAD boundaries in each cell type and compared insulation scores at these sites.

#### 2.11.3 A/B Compartment Analyses

Whole‐genome compartment analyses (PCA) on Micro‐C contact matrix at 25 kb resolution within 100 kb windows and the following PC1‐based compartment discovery were performed by HOMER. Different compartment changes were defined similarly as previous studies:^[^ ^41^, ^42^ ^]^ A to stronger A (SKBR3_HR PC1–SKBR3 PC1 > 0.2 & SKBR3 PC1 > 0.2), B to A (SKBR3_HR PC1 > 0 & SKBR3 PC1 < 0), B to weaker B (SKBR3_HR PC1–SKBR3 PC1 > 0.2 & SKBR3_HR PC1 < −0.2), B to stronger B (SKBR3_HR PC1–SKBR3 PC1 < −0.2 & SKBR3 PC1 < −0.2), A to B(SKBR3_HR PC1 < 0 & SKBR3 PC1 > 0), A to weaker A (SKBR3_HR PC1 – SKBR3 PC1 < −0.2 & SKBR3 PC1 > 0.2), and the rest were all considered as stable. Saddle plots were generated with cooltools on Micro‐C contact matrix of 25 kb resolution similar as earlier works.^[^ ^41^, ^43^ ^]^ In brief, compartment score of each bin was corrected by GC densities to generate observed/expected (O/E) contact maps, followed by sorting of both rows and columns as increasing O/E scores. Rows and columns of contact maps were equally aggregated into 50 bins after removing top and bottom 2.5% of the whole‐genome eigenvector to generate the final matrices.

#### 2.11.4 ABC and ABC‐P^2^ Scores

ABC score was suggested to quantify the cooperative activation effects of enhancers on their target promoters by integrating both enhancer activity and promoter‐enhancer contacts within a certain distance, which could be calculated according to previous studies.^[^ ^41^, ^44^ ^]^ For those active enhancers, which are defined as significant enrichments of H3K27ac peaks discovered in ±1 Mb area of s specific gene promoter, their H3K27ac signals were quantified and normalized as A scores. The promoter‐enhancer contact C score presents the normalized contacts between a certain promoter and its potential enhancers in 5 kb resolution within the ±1 Mb area. For the ABC score of each promoter, the A score of each enhancer was multiplied by its corresponding C score, and a summation was made if multiple enhancers existed. The p value of each promoter was defined as the calibrated signal of H3k4me3 within the ±2.5 kb area around the transcriptional start site (TSS). And ABC‐P^2^ score was calculated as the multiplication of the ABC score of a certain gene with the square of its P value.

### 2.12 Statistical Analysis

Statistical analyses were performed with GraphPad Prism and R. For boxplots, center lines represent median values, box limits represent the 25^th^ and 75^th^ percentiles, while whiskers indicate 1.5 times the interquartile range (IQR) from the 25th and 75th percentiles. For bar and other plot types, means ± standard deviation was indicated above. Student's test was applied for comparison between two groups. *p* < 0.05 was considered as limit of significance and marked as ^*^ *p* < 0.05, ^**^ *p* < 0.01, ^***^ *p* < 0.001. Three biological repeats were applied for RNA‐seq, while at least two biological repeats were applied for CUT&Tag and Micro‐C sequencing.

## 3 Results

### 3.1 Variation of Gene Expression and Metabolic Reprogramming Accompanied Secondary Trastuzumab Resistance Formation

**Figure 1:**
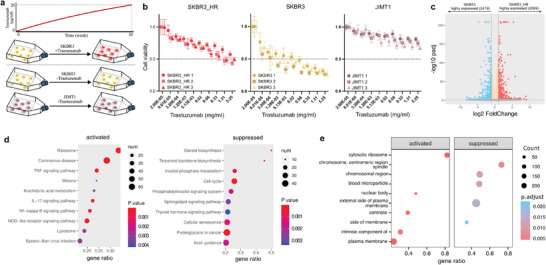
Establishment of secondary trastuzumab‐resistant cells. a) Experimental design for the establishment of secondary trastuzumab‐resistant cell. SKBR3 cells were treated with trastuzumab from 1 to 20 ug ml^−1^ concentration in 30 weeks period, while SKBR3 and JIMT1 cells were cultured in normal conditions for 30 weeks were considered as control. b) Viability of three cell types in different trastuzumab concentrations. c) Variant expression of genes during secondary trastuzumab‐resistance formation (Padj < = 0.05 & |log2 foldchange|> = 0.5 is defined as significant change). d) KEGG pathway enrichment of upregulated and downregulated genes during secondary trastuzumab‐resistance formation. e) GO cellular component enrichment of upregulated and downregulated genes during secondary trastuzumab‐resistance formation.

To induce adaptation of HER2‐positive breast cancer to trastuzumab, we exposed trastuzumab‐sensitive HER2‐positive breast cancer cells (SKBR3) to gradually increasing concentrations of trastuzumab over a 30‐week period, starting from 1 µg ml^−1^ and reaching a final concentration of 20 µg ml^−1^ (**Figure** **1**a). Both cell types underwent cell line authentication based on short tandem repeats (STR). While the trastuzumab resistance observed in the final SKBR3_HR cells may not have been as robust as that seen in the primary trastuzumab‐resistant HER2‐positive JIMT1 cells, a significant improvement was evident compared to the original SKBR3 cells. Notably, the IC50 (half‐maximal inhibitory concentration) increased from less than 1.28 µg ml^−1^ to nearly 5.25 mg ml^−1^ (Figure 1b).

During the formation of trastuzumab resistance, we observed significant variations in global gene expression, with good interclass consistency demonstrated in three independent repeats of each cell type (Figure S1a, Supporting Information). In more detail, we identified 2479 downregulated genes and 2069 upregulated genes (Figure 1c). The enhanced expression of ribosomal and lysosomal components may indicate improved protein synthesis and cell component recycling. Additionally, the upregulation of genes associated with TNF, IL‐17, and NOD pathways suggests potential alterations in anti‐tumor immune responses (Figure 1d,e). Conversely, the downregulation of chromosomal proteins, particularly those associated with spindle formation, along with reduced cell cycle activity, may regulate the rate of cell proliferation and contribute to drug resistance (Figure 1d,e; Figure S1b, Supporting Information).

### 3.2 Altered Lipid Metabolism Affects Cell Properties

Metabolic reprogramming is a widely observed phenomenon in cancer and is considered a key driver of tumor development. To investigate how altered gene expression impacts cellular metabolism, we calculated the relative activity scores of major metabolic pathways in both SKBR3 and SKBR3_HR cells using an algorithm based on transcriptomic data, as suggested by Xiao et al.^[^ ^39^ ^]^ Our analysis revealed that alterations had occurred in most of these 79 major metabolic pathways, encompassing several pathways related to carbohydrates, amino acids, and lipids (Figure S1d, Supporting Information).

**Figure 2:**
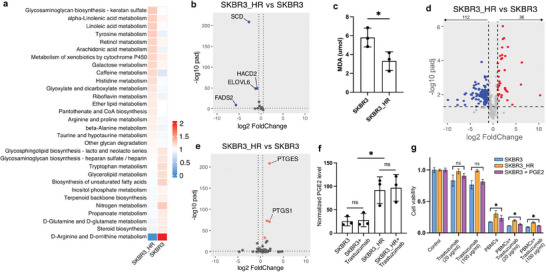
Variations of cellular metabolism during trastuzumab adaptation. a) Metabolism pathways with high different scores between SKBR3_HR and SKBR3 cells. b) Altered gene expression of unsaturated fatty acid biosynthesis pathway between SKBR3_HR and SKBR3 cells. Red dots represent significant upregulation, blue dots represents significant downregulation (Padj < = 0.05 & |log2 foldchange|> = 0.5 is defined as significant change). c) MDA values of SKBR3_HR and SKBR3 cells. d) Altered levels of free unsaturated fatty acids and lipids containing UFAs between SKBR3_HR and SKBR3 cells. Red dots represent significant upregulation, blue dots represents significant downregulation (Padj < = 0.05 & |log2 foldchange|> = 1 is defined as significant change). e) Altered gene expression of arachidonic acid metabolism pathway between SKBR3_HR and SKBR3 cells. Red dots represent significant upregulation, blue dots represents significant downregulation (Padj < = 0.05 & |log2 foldchange|> = 0.5 is defined as significant change). f) Extracellular prostaglandin E2 level of SKBR3_HR and SKBR3 cells with or without trastuzumab treatment. g) Different cell viabilities of breast cancer cells treated with PBMCs and different concentrations of trastuzumab with or without additional PGE2 supplement. The situations from left to right are control, 20 µg ml^−1^ trastuzumab, 100 µg ml^−1^ trastuzumab, PBMCs, PBMCs + 20 µg ml^−1^ trastuzumab, and PBMCs + 100 µg ml^−1^ trastuzumab.

By filtering the pathways based on a difference greater than 0.1 between the two cell types, we identified several metabolism pathways that underwent more significant changes during the development of secondary trastuzumab resistance (**Figure** **2**a). Among these, metabolic pathways involving amino acids such as tyrosine, histidine, arginine, and proline, as well as carbohydrates like galactose, exhibited activation. Additionally, pathways related to unsaturated fatty acids (UFAs) such as α‐linoleic acid, linoleic acid, and arachidonic acid showed increased activity. In contrast, the biosynthesis of UFAs and steroids, as well as the metabolism of certain D‐type amino acids, were suppressed.

The abnormal products of altered lipid metabolism can influence the activities of both cancer cells and TME components. During the formation of resistance, the downregulation of SCD, FADS2, and HACD2 emerged as the primary reason for suppressing UFAs synthesis in SKBR3_HR cells (Figure 2b). This reduction in global UFA levels, confirmed by the decrease in the MDA value (Figure 2c), led to a significant reshaping of cellular fatty acid composition. Reducing SCD level in SKBR3 cells could lead to a decrease MDA value (Figure S1m,n, Supporting Information). Specifically, more than 110 UFAs or UFAs‐containing lipids exhibited decreases, while only 36 showed increases (Figure 2d). Furthermore, among the activated lipid metabolism pathways, we observed that the conversion of arachidonic acid to prostaglandin E2 (PGE2) (Figure S1e, Supporting Information) was stimulated by the upregulation of two specific genes, PTGS1 and PTGES (Figure 2e). PGE2 is widely recognized as an immune inhibitor, and the increased production of PGE2 by SKBR3_HR cells (Figure 2f) has the potential to diminish the anti‐tumor activities of immune cells even with trastuzumab treatment (Figure 2g). This effect may contribute to a heightened trastuzumab‐resistant ability by interfering with natural killer cell mediated ADCC or macrophage mediated ADCP.^[^ ^45^, ^46^ ^]^ And the impact of PTGS1 and PTGES on PGE2 accumulation were also observed in SKBR3 cells with overexpressed genes (Figure S1o,p, Supporting Information).

Additionally, we noted the downregulation of SCD, coupled with the upregulation of PTGS1 and PTGSE, in tumor samples from some HER2‐positive breast cancer patients exhibiting secondary trastuzumab resistance compared to trastuzumab‐sensitive patients. This finding aligns with our discoveries at the cellular level (Figure S1q, Supporting Information).

### 3.3 Reshaping of Promoter Epigenetic Modifications and Active Enhancers

While several genetic and epigenetic variations are believed to occur during the adaptation of cancer cells to anti‐cancer drugs, we posit that the trimethylations of histone H3K27 and H3K4 at promoter regions may be more flexible factors that drive global variations in gene expression.

The overall levels of H3K27me3 and H3K4me3 showed downregulation during the formation of trastuzumab resistance (Figure S2a–c, Supporting Information). To precisely identify the altered positions of H3K27me3 and H3K4me3, we conducted CUT & Tag using three replicates of SKBR3 and SKBR3_HR cells, which displayed good interclass correlations (Figure S2e,f, Supporting Information). Our analysis of the distribution of H3K27me3 and H3K4me3 signals in 25 kb bins across the whole genome revealed reductions in both levels in SKBR3_HR cells, consistent with the western blot results (Figure S2h,i, Supporting Information).

**Figure 3:**
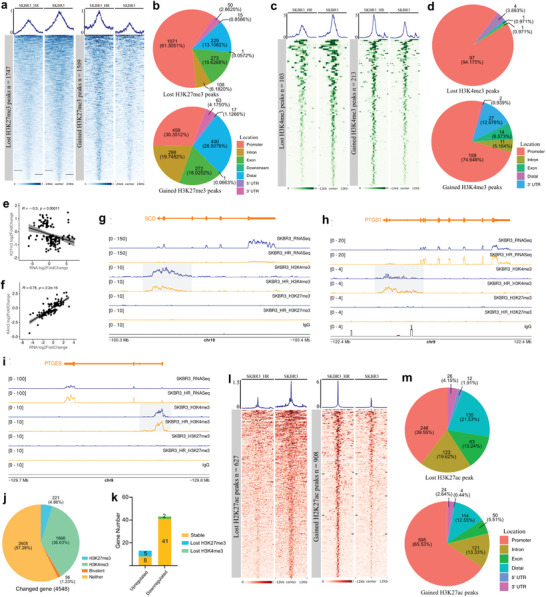
Altered Histone modifications during trastuzumab adaptation a) Altered H3K27me3 peaks during secondary trastuzumab‐resistance formation. b) Distributions of lost and gained H3K27me3 peaks during secondary trastuzumab‐resistance formation. c) Altered H3K4me3 peaks during secondary trastuzumab‐resistance formation. d) Distributions of lost and gained H3K4me3 peaks during secondary trastuzumab‐resistance formation. e) The Pearson correlation coefficients between log2 fold changes of promoter H3K27me3 level and expression of significantly altered genes (R represents correlation coefficient, *p* represents significant value). f) Pearson correlation coefficients between log2 fold changes of promoter H3K4me3 level and expression of significantly altered genes (R represents correlation coefficient, *p* represents significant value). g–i) RNA‐seq and CUT & Tag tracks at three gene locus (SCD, PTGS1 and PTGES) in SKBR3_HR and SKBR3 cells. Grey areas indicate the altered H3K4me3 peaks at promoter regions. j) Promoter modification types of altered genes in SKBR3 cells. k) Modification changes at bivalent promoters of altered genes during secondary trastuzumab‐resistance formation. l) Altered H3K27ac peaks during secondary trastuzumab‐resistance formation. m) Distributions of lost and gained H3K27ac peaks during secondary trastuzumab‐resistance formation.

In total, we detected 9610 and 8946 H3K27me3 peaks in SKBR3 and SKBR3_HR cells, with the majority distributed in promoters, introns, and distal regions (Figure S3a, Supporting Information). Among them, 6779 H3K27me3 peaks were conserved in both cell types. However, 1747 peaks were mainly enriched in promoters, introns, exons, and distal regions and were lost, while 1509 peaks in similar regions were gained during trastuzumab resistance formation (**Figure** **3**a,b; Figure S3b, Supporting Information). Additionally, we confirmed 10 042 and 8264 H3K4me3 peaks in SKBR3 and SKBR3_HR cells, with the majority abundant in promoters (Figure S3c, Supporting Information). Among these, 9559 H3K4me3 peaks remained stable in both cell types. However, 103 lost peaks were predominantly located in promoters, and 213 gained peaks were distributed not only in promoters but also in other gene regions (Figure 3c,d; Figure S3d, Supporting Information).

We observed alterations in H3K27me3 peaks at the promoters of genes related to nerve cell activities, caffeine response, and iron transport (Figure S3e,f, Supporting Information). Promoters with altered H3K27me3 peaks were primarily associated with genes enriched in molecular transport, catabolic processes, ERK or MAPK signaling, and prostanoids‐related metabolic pathways (Figure S3g,h, Supporting Information).

We also examined the correlations between significant changes in gene expression and the alterations in their promoter H3K27me3 and H3K4me3 levels. We found that H3K27me3 displayed a weak negative correlation with gene expression, while H3K4me3 revealed a strong positive correlation (Figure 3e,f). This suggests that promoter H3K4me3 plays a more crucial role in driving altered gene expression than the repressive function of H3K27me3 during the formation of trastuzumab resistance. Notably, during the formation of trastuzumab resistance, the promoter regions of crucial genes involved in UFAs synthesis (SCD) and arachidonic acid metabolism (PTGS1 and PTGES) exhibited significant changes in H3K4me3 signals rather than H3K27me3 (Figure 3g–i; Figure S3i, Supporting Information). This specific pattern of alterations underscores their regulatory role in lipid metabolic reprogramming.

Bivalent promoters are characterized by the simultaneous co‐localization of both H3K27me3 and H3K4me3 modifications, which allows for rapid and precise transcriptional regulation.^[^ ^47^ ^]^

To uncover whether bivalent promoters contribute to gene expression during trastuzumab resistance formation, we examined the distributions of H3K27me3 and H3K4me3 signals at promoter regions in both SKBR3 and SKBR3_HR cells.

In SKBR3 cells, more than 70% of total gene promoters had neither H3K27me3 nor H3K4me3 peaks, while 21.22% and 5.21% possessed H3K4me3 or H3K27me3 peaks, respectively. Only approximately 220 gene promoters exhibited both types of modifications (Figure S4a, Supporting Information). Among the altered genes, more than half of them lacked significant modifications at their promoter regions, with 36.63% and 4.86% having H3K4me3 or H3K27me3 peaks, respectively. Additionally, 56 gene promoters displayed bivalent modifications (Figure 3j).

In detail, we observed that 13 genes were upregulated during trastuzumab resistance formation. Among these, 4 showed a loss of promoter H3K27me3 peaks, while the rest remained stable. Additionally, 43 genes were downregulated, with 2 of them displaying a loss of H3K4me3 peaks, while the remainder retained stable modifications (Figure 3k).

The expression of FAM83A, considered a potential biomarker in lung and bladder cancers as well as contributed to EGFR‐TKI resistance,^[^ ^48^, ^49^, ^50^ ^]^ was activated in SKBR3_HR cells, accompanied by a significant loss of H3K27me3 but stable H3K4me3 signals at promoter regions (Figure S4c, Supporting Information). In contrast, the PTPRM gene, which exhibits low expression in cancers like glioblastoma multiforme and is associated with increased cell migration,^[^ ^51^, ^52^ ^]^ was suppressed in SKBR3_HR cells, with a dramatic reduction in promoter H3K4me3 but steady H3K27me3 (Figure S4d, Supporting Information).

In addition to histone modifications in promoter regions, it's worth noting that non‐promoter H3K27ac peaks, typically defined as active enhancer regions, also play indispensable roles in regulating gene expression.^[^ ^53^ ^]^

CUT & Tag was also employed to map H3K27ac signals in both SKBR3 and SKBR3_HR cells, demonstrating good interclass consistency (Figure S2g, Supporting Information). We observed an overall upregulation in H3K27ac levels during the formation of trastuzumab resistance, a finding that was further confirmed by western blot analysis and whole‐genome H3K27ac signal distribution plots (Figure S2a,d,j, Supporting Information).

We identified a total of 21 325 H3K27ac peaks in SKBR3 cells and 21 606 H3K27ac peaks in SKBR3_HR cells, primarily located in promoters, introns, and exons (Figure S5a, Supporting Information). During the formation of trastuzumab resistance, 20 698 H3K27ac peaks remained stable, while 627 were lost, and 908 were gained (Figure S5b, Supporting Information; Figure 3l)

In both SKBR3 and SKBR3_HR cells, 10 955 and 10 889 H3K27ac peaks, respectively, out of promoters were considered active enhancer regions.^[^ ^54^ ^]^ Consequently, during resistance formation, 10 576 active enhancers were shared between both cell types. Meanwhile, 379 enhancers became silent, and 313 transitioned into an active state (Figure S5c, Supporting Information).

### 3.4 Rearranged Genome‐Wide Interactions Regulate Gene Expression

Rearrangement of 3D genome architecture has been shown to accompany breast cancer progression and responses to anti‐cancer drugs.^[^ ^34^, ^55^, ^56^, ^57^ ^]^ To investigate how genome architecture changes and its impact on gene expression, we employed Micro‐C, which utilizes MNase instead of restriction endonuclease (RE) to obtain more evenly sized DNA fragments and achieve higher resolution for local interactions than standard Hi‐C protocol. We applied this method to both SKBR3 and SKBR3_HR cells, and the results demonstrated high concordance between two replicates (Figure S6a,b, Supporting Information).

**Figure 4:**
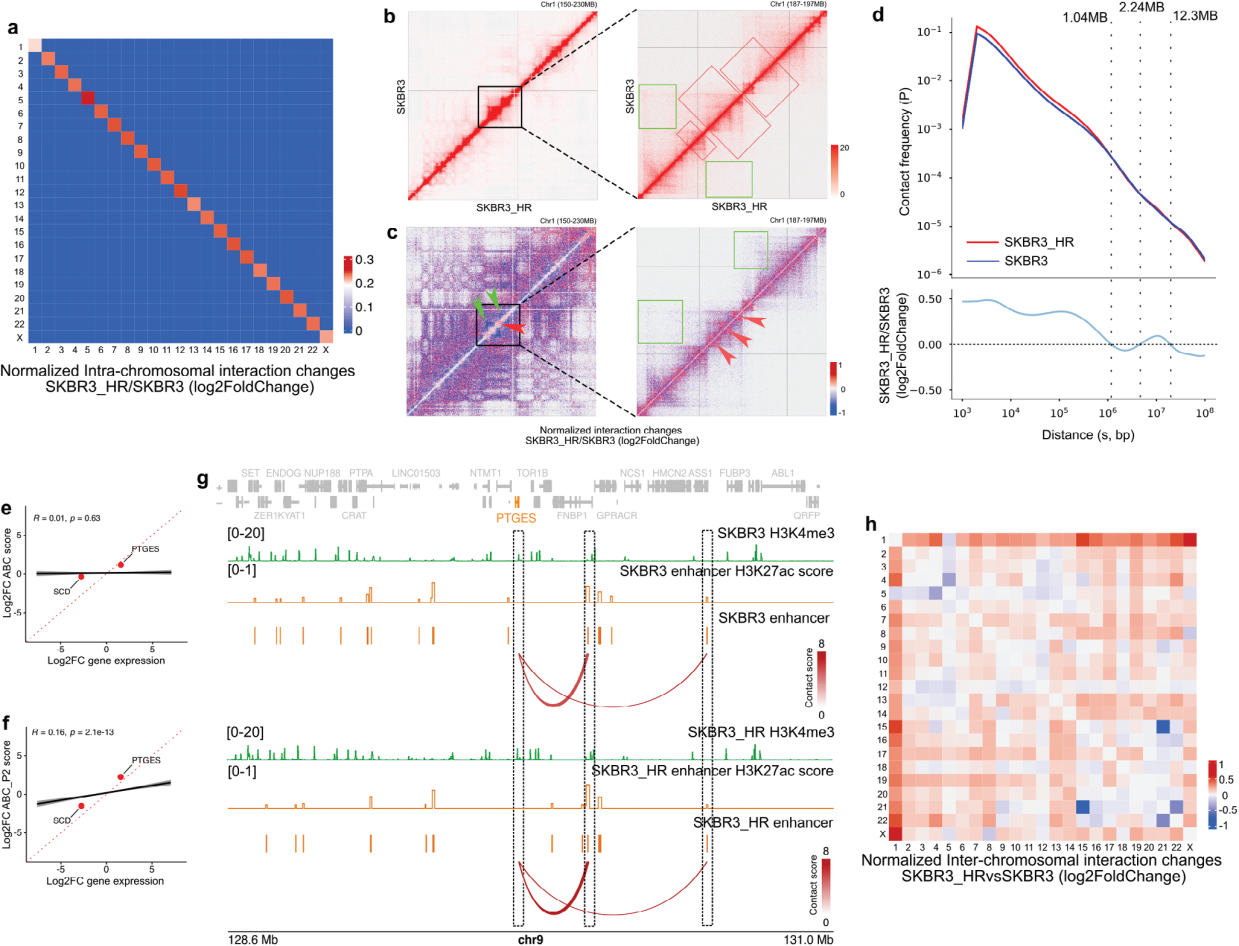
Variable intra‐ and inter‐chromosomal interactions during trastuzumab adaptation. a) Log2 fold changes of intra‐chromosomal interactions (SKBR3_HR/SKBR3 cells). b) Detailed intra‐chromosomal interactions in certain chromosomal regions (chr1: 150–230 and 187–197 MB) of SKBR3_HR and SKBR3 cells. Green squares indicate lost interactions, red squares indicate gained interactions. c) Log2 fold changes of intra‐chromosomal interactions of chromosome 1 fragment in (b) (SKBR3_HR/SKBR3 cells). Green squares indicate reduced interactions, red arrows indicate enhanced interactions. d) Top: Relationship between contact frequency (P) of intra‐chromosomal interactions and genomic distance (s) in both SKBR3_HR (red) and SKBR3 (blur) cells. Bottom: Log2 fold changes of contact frequency ranked by distances (SKBR3_HR/SKBR3). The dotted lines represent the crossing points of two curves (1.04, 2.24, and 12.3 MB). e,f) ABC and ABC‐P^2^ scores of specific genes (PTGES and SCD), R and *p* values reveal the global Pearson correlation coefficients mentioned in Figure S6e,f (Supporting Information). g) Track plot showing the distributions of H3K4me3 peaks and enhances in ± 1 MB regions around PTGES gene. The curve below links PEGES promoter and corresponding enhances and indicates the contact scores between them. The dashed boxes indicates the promoter regions of PTGES as well as enhancers in contact in SKBR3 and SKBR3_HR cells. h) Log2 fold changes of inter‐chromosomal interactions (SKBR3_HR/SKBR3 cells).

Micro‐C data indicated widespread changes in genomic interactions during the formation of secondary trastuzumab resistance. In general, intra‐chromosomal interactions within all 22 and X chromosomes were found to be strengthened (**Figure** **4**a).

We observed that more short‐range contacts were established in SKBR3_HR cells, as indicated in Figure 4b (red boxes). In contrast, long‐range contacts showed a decrease, as shown in Figure 4b (green boxes). By calculating the log2 fold changes in interactions, we can more clearly demonstrate the reduction of short‐range interactions (Figure 4c, green arrows and boxes) and the increase in long‐range interactions (Figure 4c, red arrows). Similar patterns were also observed in other chromosome regions (Figure S6c, Supporting Information).

A Contact frequency (P) versus Distance (s) curve revealed that during resistance formation, global inter‐chromosomal short‐distance contacts (<1.04 MB) showed a dramatic upregulation, while there was a slight increase in mild‐to‐long‐distance contacts (≈ 2.24–12.3 MB). In contrast, short‐to‐mild‐distance contacts (≈ 1.04–2.24 MB) as well as long‐range contacts (> 12.3 MB) exhibited slight and partial downregulation, respectively (Figure 4d).

The coordination between promoters and enhancers established through intra‐chromosomal contacts, along with the activities of both promoters and enhancers themselves, likely plays a critical role in regulating gene expression.

When we simply assess how intra‐chromosomal interactions and enhancer strengthens affect gene transcriptional activity, we used the scores of the activity‐by‐contact (ABC) algorithm (Figure S6d, Supporting Information). However, it is challenging to discern their global correlations (Figure S6e, Supporting Information). Furthermore, when we considered the cooperative effect between promoter activity and promoter‐enhancer contacts by modifying the ABC algorithm with additional promoter H3K4me3 signals (Figure S6d, Supporting Information),^[^ ^41^ ^]^ we obtained a series of revised ABC‐P^2^ scores, which exhibited weak correlation coefficients with gene expression (Figure S6f, Supporting Information).

Specifically, although the number and position of enhancers located within a ±1 MB genomic region around the PTGES gene were similar in SKBR3 and SKBR3_HR cells, most of them did not establish contacts with the PTGES promoter, except for two enhancers located downstream of the PTGES gene. In SKBR3_HR cells, both of these enhancers exhibited higher H3K27ac scores and promoter‐enhancer scores, while the PTGES promoter had a higher H3K4me3 score, resulting in improved ABC and ABC‐P^2^ scores (Figure 4g). However, for the SCD gene, the strengths of its relevant enhancers, promoter‐enhancer contacts, as well as promoter H3K4me3 signals, were all weaker in SKBR3_HR cells compared to SKBR3 cells, leading to lower ABC and ABC‐P^2^ scores (Figure S6g, Supporting Information).

Although the overall inter‐chromosomal contacts had a slight reduction in SKBR3_HR cells (Figure S6h, Supporting Information), the actual situation appeared more complex. As indicated in Figure 4h, both upregulations and downregulations co‐existed: most chromosome 1‐relevant contacts were enhanced, except with chromosome 5, while significant reductions in interactions occurred between chromosomes 4 and 5, chromosomes 15 and 21, as well as chromosomes 21 and 22.

More specifically, gained and lost inter‐chromosomal contacts were enriched in certain genomic regions. For example, the top 0.5% of the most significantly increased contacts between chromosomes 1 and X were concentrated in chr1: 60–80 MB and chr X: 62–75 MB. Meanwhile, the top 0.5% of the most significantly decreased contacts between chromosomes 4 and 5 were concentrated in chr 4: 52–76 MB and chr 5: 4–46 MB (Figure S6i,j, Supporting Information). This enrichment of inter‐chromosomal contacts may play particular regulatory roles in gene expression.^[^ ^58^, ^59^ ^]^

Chromatin loops and topological‐associated domains (TADs) are even more complex 3D genomic architectures that go beyond genome contacts and hold greater structural and functional significance.^[^ ^60^ ^]^

Chromatin loops, often representing high‐frequency interactions between chromatin regions, are sometimes referred to as “significant interactions.” During resistance formation, we observed that the number of chromatin loops in SKBR3_HR cells had increased by nearly onefold compared to SKBR3 cells, indicating a global increase in intra‐chromosomal interactions as well (Figure S7a, Supporting Information). Furthermore, we noted that the distribution of loop sizes in SKBR3_HR cells was smaller than in SKBR3 cells, also mirroring the distribution of intra‐chromosomal contacts and indicating an increase in interactions between nearby chromatin regions (Figure S7b, Supporting Information).

TADs, which define chromatin regions with more inter‐chromosomal contacts within them than with their surrounding regions, play a crucial role in genome organization.^[^ ^61^ ^]^ We observed that, despite the insulation scores of all TAD boundaries remaining relatively stable during resistance formation (Figure S7c, Supporting Information), there were similar total numbers of TADs in both SKBR3 and SKBR3_HR cells (Figure S7d, Supporting Information). Notably, the overall size of TADs in SKBR3_HR cells was smaller than in SKBR3 cells (Figure S7e, Supporting Information), also indicating an increase in short‐distance contacts. Furthermore, the top 5% of TADs that changed during resistance formation displayed an increase in CTCF but a decrease in the cohesin member Rad21 at their boundaries (Figure S7f,g, Supporting Information).

When comparing SKBR3 and SKBR3_HR cells, we observed stable TADs but lost chromatin loops around the SCD gene loci (Figure S7h, Supporting Information). Conversely, there were rearranged TADs and chromatin loops around the PTGS1 gene loci in SKBR3_HR cells (Figure S7j, Supporting Information).

### 3.5 Altered Compartment A/B Links Histone Modifications

Principal component analysis (PCA) was applied to segregate the genome into two primary compartments based on Micro‐C data. Of them, compartment A typically represents the “active” chromatin regions, while compartment B signifies the “inactive” chromatin regions.^[^ ^60^ ^]^

**Figure 5:**
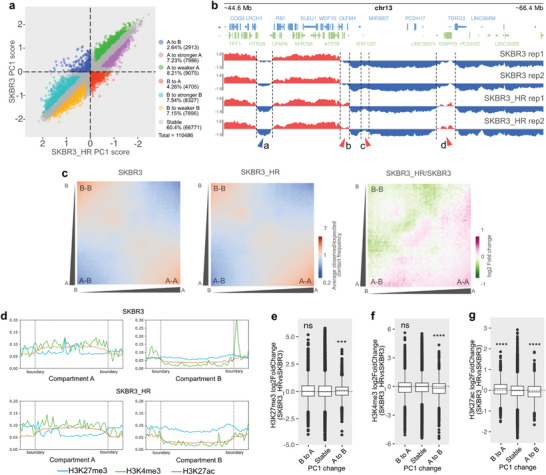
Altered A/B compartments during trastuzumab adaptation. a) The distributions of compartment scores at 25 kb genomic bin resolution in SKBR3_HR and SKBR3 cells. 7 colors represent different categories of bins based on their score changes (A to B, B to A, A to stronger A, A to weaker A, B to stronger B, B to weaker B, and stable). b) PCA tracks at certain chromosomal region (chr13: 44.6–66.4 MB) of both SKBR3 and SKBR3_HR cells. Blue arrow a represents B to stronger B, red arrow b and d represent B to A, and red arrow c represents B to weaker B. c) Saddle plots indicate the compartment interactions of SKBR3 and SKBR3_HR cells. Color scales represent observed/expected (O/E) contact frequencies (left and middle), and log2 fold change of O/E contact frequencies (right). d) Distributions of three histone modifications (H3K27me3, H3K4me3, and H3K27ac) across compartment A and B in SKBR3 and SKBR3_HR cells. e–g) Distribution of log2 histone modifications changes (H3K27me3, H3K4me3 and H3K27ac) in different types of compartment changes (B to A, stable and A to B).

During trastuzumab resistance formation, approximately 40% of compartments exhibited significant variations in the 25 kb‐binned genome matrices. These variations included conversions between different compartment types (A to B and B to A) as well as changes within the same compartment type (A to stronger A, A to weaker A, B to stronger B, and B to weaker B) (**Figure** **5**a). Specifically, B to A conversions occurred in 4.26% of all genome bins, which was almost twice as frequent as A to B conversions, while other types of changes each affected nearly 7% to 8% of total bins.

In a specific genomic region (chr13: 44.6–66.4 MB), we observed three different types of compartment changes (B to A, B to stronger B, and B to weaker B) during the development of trastuzumab adaptation (Figure 5b). Furthermore, genome‐wide inter‐compartment contacts, as depicted in saddle plots, revealed an increase in A–A interactions but a decrease in B–B interactions during the adaptation process (Figure 5c).

Active and suppressive histone modifications across the entire genome are often associated with the distribution of A/B compartments.^[^ ^43^, ^62^ ^]^ In both SKBR3 and SKBR3_HR cells, we observed that H3K4me3 and H3K27ac peaks were more concentrated in compartment A, while H3K27me3 signals were enriched in compartment B (Figure 5d). Additionally, we noted that the differences in H3K4me3 between compartments A and B were larger than those in H3K27ac.

Altered H3K27me3, H3K4me3, and H3K27ac modifications were all associated with compartment conversions during trastuzumab resistance formation, although they contributed differently to this process. During the conversion from B to A compartments, H3K27me3 and H3K4me3 signals remained almost unchanged, while H3K27ac levels increased. In contrast, the transition from A to B compartments was accompanied by increased H3K4me3 and H3K27me3 levels but decreased H3K27ac levels (Figure 5e–g).

In detail, we observed that the most significant changes in H3K27me3 and H3K4me3 were concentrated in the top 30% of A to B compartment conversions (Figure S8a,b, Supporting Information). Alterations in H3K27ac were distributed in both B to A and A to B changes (Figure S8c, Supporting Information), and the enrichment of H3K27ac‐based enhancers also increased in B to A compartments (Figure S8d, Supporting Information).

Furthermore, within the same compartment type, A to stronger A and B to weaker B changes showed trends of activation, while A to weaker A and B to stronger B indicated trends of suppression. H3K27me3 signals exhibited decreased enrichment during A to weaker A and increased enrichment during B to weaker B (Figure S8e, Supporting Information). H3K4me3 signals were notably suppressed during B to stronger B (Figure S8f, Supporting Information), and H3K27ac signals showed increased concentration in A to stronger A while decreased concentration in B to stronger B (Figure S8g, Supporting Information).

## 4 Discussion

Differing from primary drug resistance, which arises from random evolutionary processes, secondary drug resistance can be viewed as a targeted evolutionary response to selective pressure.^[^ ^63^, ^64^, ^65^, ^66^ ^]^ In contrast to the direct cytotoxic effects of traditional chemotherapy drugs, which encompass various mechanisms such as DNA damage, cell cycle arrest, and inhibition of cell division, capable of swiftly eliminating cancer cells,^[^ ^67^ ^]^ the primary function of trastuzumab is to prevent HER2 dimerization and the subsequent activation of downstream signaling cascades that promote cell proliferation. This milder anti‐tumor effect affords breast cancer cells ample time to develop adaptations, rather than undergoing the more rigorous Darwinian selection seen during the formation of chemoresistance.

Presently, research related to trastuzumab resistance predominantly focuses on structural and expression variations, as well as the abnormal activation of downstream or bypass signaling pathways that originate from HER2, but often pays less attention to other cellular processes. While we have noticed a slight downregulation of HER2 as a potential response to trastuzumab (Figure S1c, Supporting Information), our preference is to propose that there is a concurrent global metabolic reprogramming that occurs during the development of trastuzumab adaptation, particularly within pathways associated with lipid metabolism. This reprogramming may be influenced by changes in a select few genes involved in critical reactions within specific pathways. For example, the activation of arachidonic acid metabolism appears to be primarily driven by the upregulation of two PGE2 synthesis enzymes, PTGS1 and PTGES. Additionally, the suppression of unsaturated fatty acid synthesis may be constrained by the action of the fatty acid desaturase SCD.

Several studies have confirmed the detrimental effects of excessive PGE2, the products of activated arachidonic acid metabolism, in tumor progression.^[^ ^68^ ^]^ On one side, PGE2 can promote tumor cell proliferation by activating PGE2 receptors through autocrine or paracrine signaling.^[^ ^69^, ^70^ ^]^ On the other side, within TME, PGE2 serves as an immunosuppressive agent, diminishing the activities of dendritic cells, NK cells, and Th1 cells and also stimulates immunosuppressive cells such as M2 macrophages and Treg cells.^[^ ^71^, ^72^ ^]^ Additionally, PGE2 promotes angiogenesis by stimulating microvascular endothelial cells, contributing to nutritional and oxygen requirements as well as metastasis.^[^ ^73^ ^]^ Hence, inhibitors of cyclooxygenases (COX), including aspirin and other non‐steroidal anti‐inflammatory drugs, may hold promise in restricting breast cancer progression.^[^ ^74^, ^75^, ^76^ ^]^

At the same time, we have demonstrated a global reduction in UFAs, which is likely associated with the downregulation of SCD. While free UFAs did not exhibit significant variations (Figure S1f, Supporting Information), we extensively observed reductions in major membrane lipids (PC, PE, PI, PS, cardiolipins, and ceramide lipids) containing UFAs during the formation of trastuzumab resistance (Figure S1g–l, Supporting Information). This decrease in the proportion of UFAs within cell membranes may serve to reduce cellular damage caused by reactive oxygen species (ROS), providing cancer cells with greater tolerance to increased mitochondrial activity and anti‐tumor drug treatments.^[^ ^77^, ^78^ ^]^

It's important to recognize that the actual evolutionary process of trastuzumab adaptation during the clinical treatment of HER2‐positive breast cancers will differ, at least partially, from what occurs during in vitro induction. In clinical settings, cancer cells encounter a more complex array of extracellular circumstances. These environmental stresses include nutritional shortages and hypoxia, as well as direct or indirect interactions with neighboring cells such as immune cells, fibroblasts, and epithelial cells. Additionally, trastuzumab‐mediated processes like ADCC, ADCP, and potential antibody‐dependent complement activation (ADCA)^[^ ^79^ ^]^ collaborate to stimulate cancer cells to develop both metabolic and non‐metabolic survival and progression strategies.

Chemotherapy agents such as cisplatin, doxorubicin, and cyclophosphamide exert their inhibitory effects on cancer cells primarily by inducing DNA damage. Consequently, mutations or gene rearrangements during DNA repair can be the predominant driving forces behind evolutionary drug adaptation during such anti‐tumor treatments.^[^ ^9^, ^80^, ^81^ ^]^ In contrast, moderate anti‐tumor drugs like trastuzumab, which do not directly induce DNA damage, may rely more heavily on epigenetic alterations and the reshaping of higher genome architecture to drive cancer cell adaptive evolution.

DNA methylation stands out as one of the most extensively studied epigenetic alterations.^[^ ^82^, ^83^ ^]^ During both carcinogenesis and drug adaptation, both genome‐wide and site‐specific changes in DNA methylation have been proved. These alterations can lead to the activation or suppression of gene expression and contribute to genome instability, which in turn promotes chromosomal rearrangements. In contrast, modifications on histones are more dynamic and readily changeable, providing a flexible mechanism for regulating gene expression.^[^ ^35^, ^84^ ^]^ Various types of histone modifications play distinct regulatory roles. In addition to H3K27me3, H3K4me3, and H3K27ac, which are mentioned in this article, heterochromatin‐associated H3K9me3, as well as DNA repair‐relevant H3K36me3 and H4K20me3 are all have significant roles in cancer progression.^[^ ^85^, ^86^, ^87^, ^88^ ^]^

Furthermore, the co‐localization of both activated and suppressive histone modifications creates bivalent or “poised” chromatin.^[^ ^47^, ^89^ ^]^ This arrangement of epigenetic marks at promoter regions allows genes to be expressed at a relatively low level while retaining the capability for rapid activation or suppression. This mechanism proves effective not only in embryonic development but also in cancer processes, offering precise control over gene expression.^[^ ^90^, ^91^, ^92^ ^]^ We've observed that although the frequency of bivalent promoters in original SKBR3 cells is relatively small (1.33%; Figure S4a, Supporting Information), a significant number of them remain enriched in development‐related processes (Figure S4b, Supporting Information). This observation highlights the potential regulatory mechanisms of such gene groups in tumor progression.

3D genome architecture refers to the folding and organization of genomic DNA in association with proteins. While its role in transcriptional regulation has been extensively studied in the context of embryonic development, it has also gained significance in the field of cancer research.^[^ ^42^, ^93^, ^94^, ^95^ ^]^

Variant intra‐chromosomal contact is a critical component of 3D genome architecture alterations. It creates opportunities for promoters to interact with one or more distant enhancer elements through DNA loops, facilitating synergistic regulation of gene expression.^[^ ^96^ ^]^ While the number and position of enhancers remained relatively stable in SKBR3_HR, there was a global increase in intra‐chromosomal short‐distance contacts. This suggests the possibility that promoters are more frequently stimulated by elevated interactions with neighboring active enhancers, a phenomenon quantified by their ABC scores. Although the relationship between gene expression and ABC scores as a whole may not be readily apparent, we can still observe that reshaped promoter‐enhancer contacts may affect the expression of specific genes. Furthermore, alterations in overall inter‐chromosomal interactions may indicate changes in chromatin compartmentalization and the potential cooperation of multiple enhancers on different chromosomes.^[^ ^97^ ^]^ We observed overall alterations in TADs and loops, which represent significantly enriched chromatin contacts and demonstrate changes in chromatin structure, in both cell types. Specifically, in SKBR3_HR cells, one chromatin loop adjacent to the SCD gene was lost, while another loop was gained next to the PTGS1 gene within a newly formed TAD (Figure S7h,j, Supporting Information). The anchors of these two altered loops contain several potential transcription factor motifs (Figure S7i,k, Supporting Information), suggesting their potential regulatory roles in gene transcription.

We also observed changes in chromatin compartments associated with trastuzumab resistance formation. Specifically, more B‐type compartments transitioned into A‐type compartments, coinciding with the enrichment of activated epigenetic marks such as H3K4me3 and H3K27ac, and the loss of inhibitory epigenetic marks like H3K27me3. These observations are consistent across various cellular processes,^[^ ^98^, ^99^, ^100^, ^101^ ^]^ highlighting the strong connections between epigenetic modifications and chromatin states, and their pivotal role in transcriptional regulation during trastuzumab adaptation.

**Figure 6:**
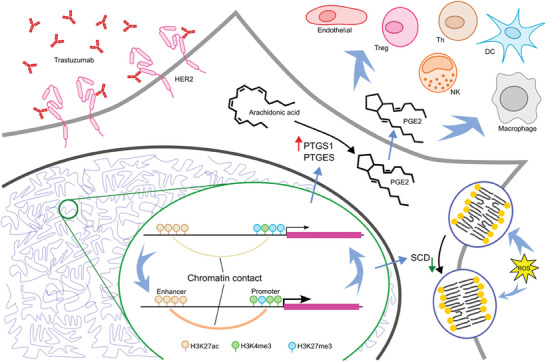
Overview of this study.

In summary, our study elucidated the metabolic reprogramming, particularly the alterations in lipid‐associated pathways, and proposed their potential impact on cancer cells and inter‐cellular interactions. Beyond this, we introduced the alternation of global H3K27me3, H3K4me3 and H3K27ac modifications as well as enhancer‐promoter contacts caused by 3D chromatin structure variation, especially highlighted their potential role in driving lipid metabolic reprogramming (**Figure** **6**). Our research opens up a promising avenue for future investigations into cancer drug resistance.

## Conflict of Interest

The authors declare no conflict of interest.

## Author Contributions

N.J.D. conceive the project. N.J.D., Y.J.H., Y.Z.H., T.Y.Z., and J.G. performed the wet lab experiments included in the manuscript. X.Q.Y. helped generate the trastuzumab‐resistant cells. N.J.D. and Y.J.H. conducted dry lab works. N.J.D. and Y.J.H. wrote the manuscript and arranged the figures. All authors reviewed the manuscript.

## Supporting information

Supporting Information

## Data Availability

The sequencing data is available in Gene Expression Omnibus (GSE244537).
