# Supplementary material for: Unveiling Alterations of Epigenetic Modifications and Chromatin Architecture Leading to Lipid Metabolic Reprogramming during the Evolutionary Trastuzumab Adaptation of HER2‐Positive Breast Cancer
Source: Adv Sci (Weinh). 2024 Mar 9;11(18):2309424. doi: 10.1002/advs.202309424 (PMC11095153; doi:10.1002/advs.202309424)
Supplement: Supplementary file 1 — Supporting Information [file ADVS-11-2309424-s001.pdf]

ADVANCED SCIENCE
Open Access

Supporting Information

for *Adv. Sci.*, DOI 10.1002/advs.202309424

Unveiling Alterations of Epigenetic Modifications and Chromatin Architecture Leading to Lipid Metabolic Reprogramming during the Evolutionary Trastuzumab Adaptation of HER2-Positive Breast Cancer

Ningjun Duan, Yijia Hua, Xueqi Yan, Yaozhou He, Tianyu Zeng, Jue Gong, Ziyi Fu, Wei Li and Yongmei Yin**

Supplementary Data

Unveiling alterations of epigenetic modifications and chromatin architecture leading to lipid metabolic reprogramming during the development of secondary trastuzumab resistance in HER2-positive breast cancer

Ningjun Duan[1,*], Yijia Hua[1], Xueqi Yan[1], Yaozhou He[1], Tianyu Zeng[1], Ziyi Fu[1], Wei Li[1], Yongmei Yin[1,*]

1. Department of oncology, First affiliation hospital of Nanjing medical university, Nanjing, 210029, China

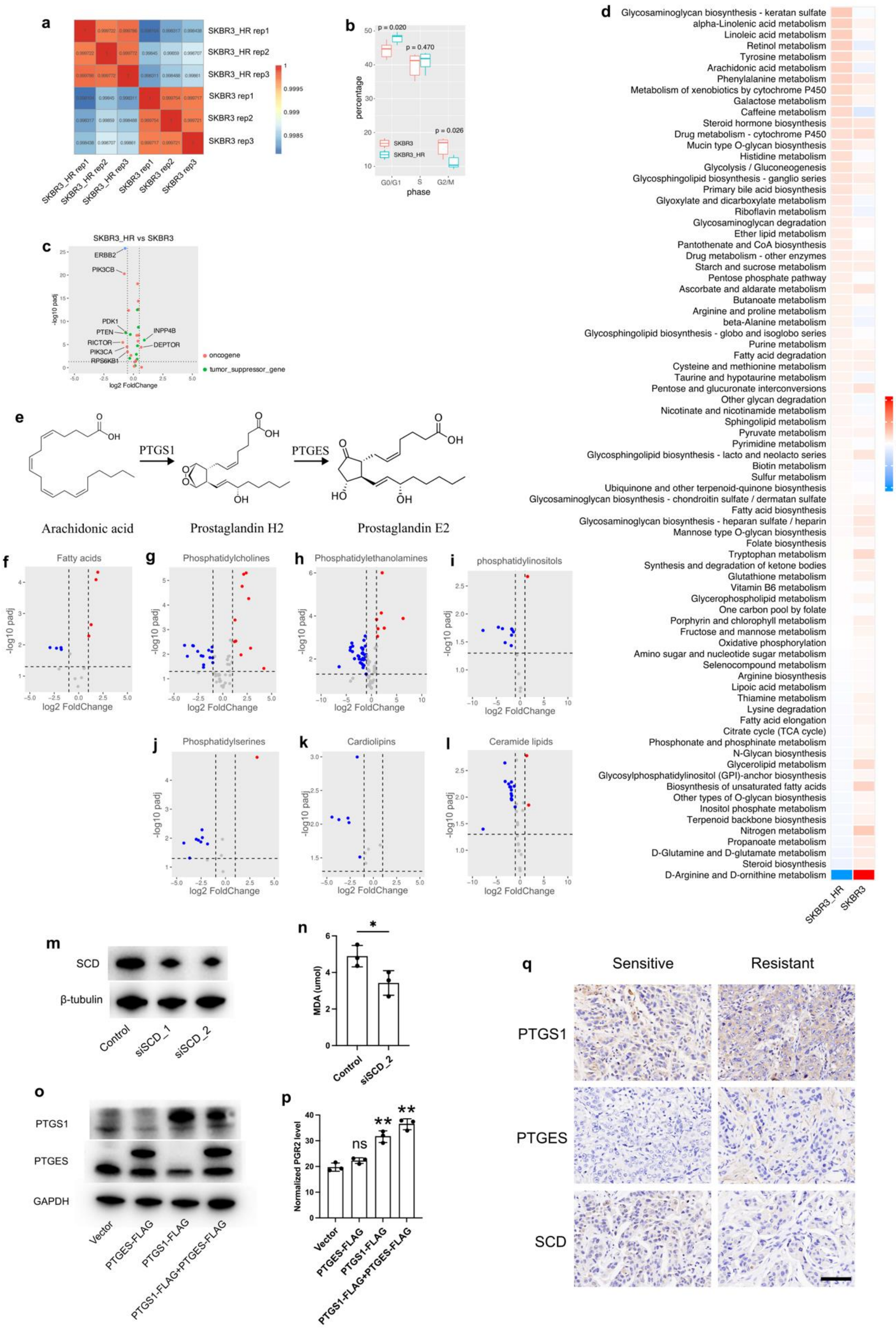

a
SKBR3_HR rep1
SKBR3_HR rep2
SKBR3_HR rep3
SKBR3 rep1
SKBR3 rep2
SKBR3 rep3
b
p = 0.020
p = 0.470
p = 0.026
percentage
SKBR3
SKBR3_HR
G0/G1
S
G2/M
phase
c
SKBR3_HR vs SKBR3
ERBB2
PIK3CB
PDK1
PTEN
RICTOR
PIK3CA
RPS6KB1
INPP4B
DEPTOR
-log10 padj
log2 FoldChange
oncogene
tumor_suppressor_gene
d
Glycosaminoglycan biosynthesis - keratan sulfate
alpha-Linolenic acid metabolism
Linoleic acid metabolism
Retinol metabolism
Tyrosine metabolism
Arachidonic acid metabolism
Phenylalanine metabolism
Metabolism of xenobiotics by cytochrome P450
Galactose metabolism
Caffeine metabolism
Steroid hormone biosynthesis
Drug metabolism - cytochrome P450
Mucin type O-glycan biosynthesis
Histidine metabolism
Glycolysis / Gluconeogenesis
Glycosphingolipid biosynthesis - ganglio series
Primary bile acid biosynthesis
Glyoxylate and dicarboxylate metabolism
Riboflavin metabolism
Glycosaminoglycan degradation
Ether lipid metabolism
Pantothenate and CoA biosynthesis
Drug metabolism - other enzymes
Starch and sucrose metabolism
Pentose phosphate pathway
Ascorbate and aldarate metabolism
Butanoate metabolism
Arginine and proline metabolism
beta-Alanine metabolism
Glycosphingolipid biosynthesis - globo and isoglobo series
Purine metabolism
Fatty acid degradation
Cysteine and methionine metabolism
Taurine and hypotaurine metabolism
Pentose and glucuronate interconversions
Other glycan degradation
Nicotinate and nicotinamide metabolism
Sphingolipid metabolism
Pyruvate metabolism
Pyrimidine metabolism
Glycosphingolipid biosynthesis - lacto and neolacto series
Biotin metabolism
Sulfur metabolism
Ubiquinone and other terpenoid-quinone biosynthesis
Glycosaminoglycan biosynthesis - chondroitin sulfate / dermatan sulfate
Fatty acid biosynthesis
Glycosaminoglycan biosynthesis - heparan sulfate / heparin
Mannose type O-glycan biosynthesis
Folate biosynthesis
Tryptophan metabolism
Synthesis and degradation of ketone bodies
Glutathione metabolism
Vitamin B6 metabolism
Glycerophospholipid metabolism
One carbon pool by folate
Porphyrin and chlorophyll metabolism
Fructose and mannose metabolism
Oxidative phosphorylation
Amino sugar and nucleotide sugar metabolism
Selenocompound metabolism
Arginine biosynthesis
Lipoic acid metabolism
Thiamine metabolism
Lysine degradation
Fatty acid elongation
Citrate cycle (TCA cycle)
Phosphonate and phosphinate metabolism
N-Glycan biosynthesis
Glycerolipid metabolism
Glycosylphosphatidylinositol (GPI)-anchor biosynthesis
Biosynthesis of unsaturated fatty acids
Other types of O-glycan biosynthesis
Inositol phosphate metabolism
Terpenoid backbone biosynthesis
Nitrogen metabolism
Propanoate metabolism
D-Glutamine and D-glutamate metabolism
Steroid biosynthesis
D-Arginine and D-ornithine metabolism
SKBR3_HR
SKBR3
e
PTGS1
PTGES
Arachidonic acid
Prostaglandin H2
Prostaglandin E2
f
Fatty acids
g
Phosphatidylcholines
h
Phosphatidylethanolamines
i
phosphatidylinositols
j
Phosphatidylserines
k
Cardiolipins
l
Ceramide lipids
-log10 padj
log2 FoldChange
m
SCD
β-tubulin
Control
siSCD_1
siSCD_2
n
MDA (umol)
Control
siSCD_2
o
PTGS1
PTGES
GAPDH
Vector
PTGES-FLAG
PTGS1-FLAG
PTGS1-FLAG+PTGES-FLAG
p
Normalized PGR2 level
ns
Vector
PTGES-FLAG
PTGS1-FLAG
PTGS1-FLAG+PTGES-FLAG
q
Sensitive
Resistant
PTGS1
PTGES
SCD

Supplementary figure 1

a, Correlations between the RNA sequencing data of SKBR3 and SKBR3_HR cells. **b,** Cell cycle distributions of SKBR3 and SKBR3_HR cells. **c,** Alternations of ERBB2(HER2) and PI3K-AKT signaling pathway members. Red dots represent inhibitors of PI3K-AKT signal pathway, blue dots represent oncogenes of PI3K-AKT signaling pathway. **d,** The overall metabolic pathway scores of SKBR3 and SKBR3_HR cells. **e,** The conversion from arachidonic acid to PGE2 and enzymes involved in. **f,** Alternations of UFAs during resistance formation. Red dots represent significant upregulation, blue dots represents significant downregulation. **g-l,** Alternations of membrane lipids containing UFAs during resistance formation. Red dots represent significant upregulation, blue dots represents significant downregulation. **m**, Knockdown of SCD in SKBR3 cells. n, MDA values of SKBR3 and SCD-knockdown SKBR3 cells. **o**, Overexpression of PTGES, PTGS1 and co-overexpression in SKBR3 cells. **p**, Extracellular prostaglandin E2 level of SKBR3 cells and SKBR3 cells with single-overexpressed or co-overexpressed PTGES and PTGS1. **q**, Immunohistochemistry(IHC) staining of PTGES, PTGS1 and SCD in tumor samples from trastuzumab seneitive and secondary resistant HER2 positive breast cancer patients (50 μm scalebar).

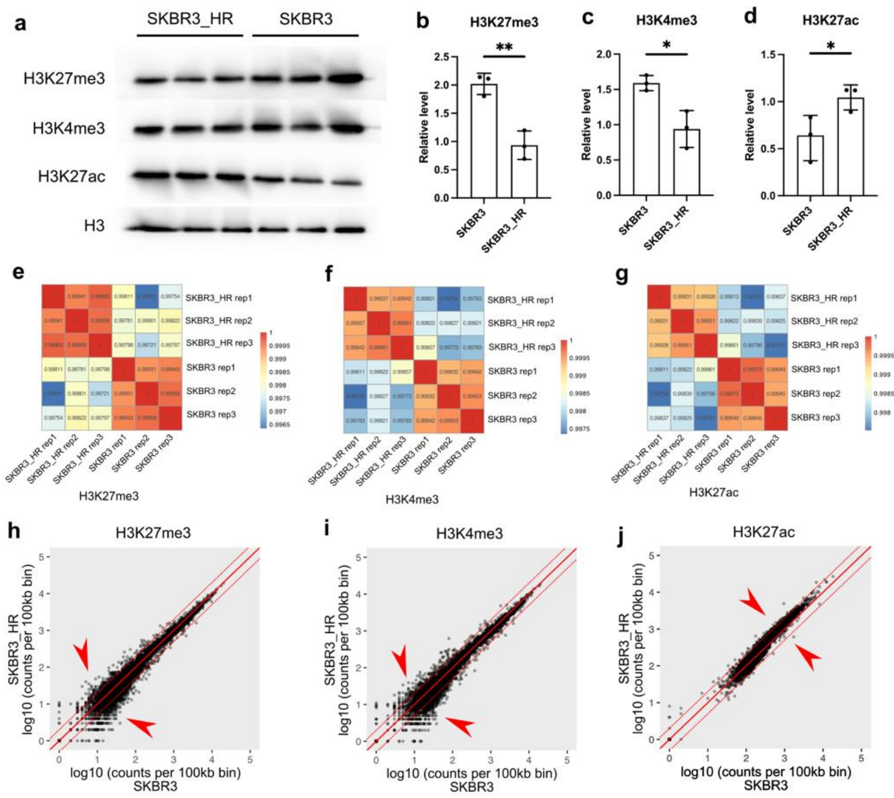

Supplementary figure 2

a, Abundances of global histone H3 modifications (K27me3, K4me3 and K27ac) in both SKBR3 and SKBR3_HR cells, and the total H3 was used as control. **b-d,** Relative abundance of three histone modifications showed in **a. e-g,** Correlations between the CUT&Tag sequencing data of three histone H3 modifications in SKBR3 and SKBR3_HR cells. **h-j,** Changes of histone H3 modification signals at 25 kb resolution for H3K27me3, H3K4me3 and H3K27ac. Two-fold change is represented by dotted lines. Arrows indicate obvious changes.

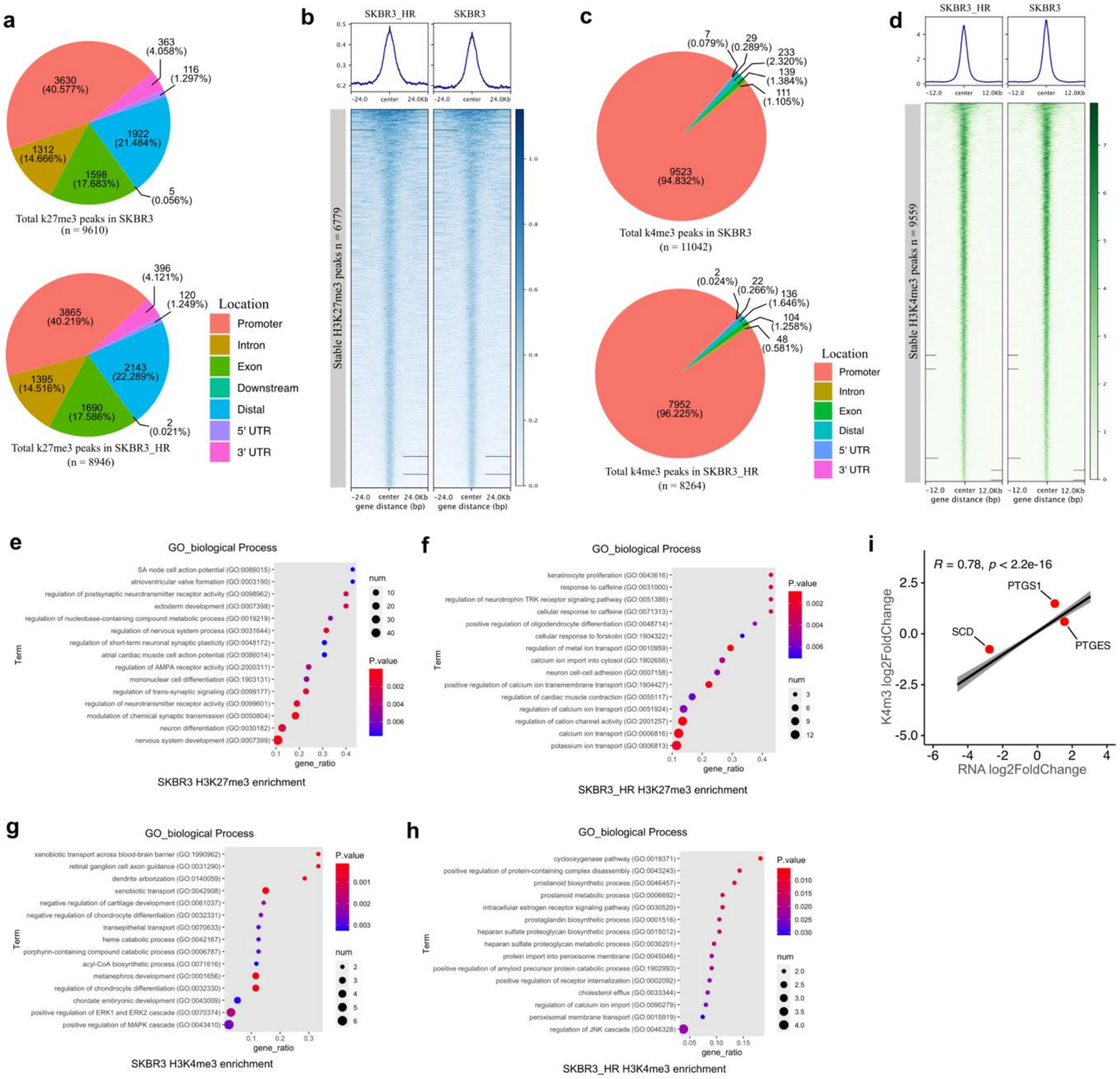

Supplementary figure 3

a, Distributions of total H3K27me3 peaks in SKBR3 and SKBR3_HR cells. **b,** Stable H3K27me3 peaks during secondary trastuzumab-resistance formation. **c,** Distributions of total H3K27me3 peaks in SKBR3 and SKBR3_HR cells. **d,** Stable H3K4me3 peaks during secondary trastuzumab-resistance formation. **e and f,** GO biological processes enrichment of gained and lost H3K27me3 peaks during secondary trastuzumab-resistance formation. **g and h,** GO biological processes

enrichment of gained and lost H3K4me3 peaks during secondary trastuzumab-resistance formation. **i,** Pearson correlation between changes of gene expression (SCD, PTGS1 and PTGES) and promoter H3K4me3 modifications.

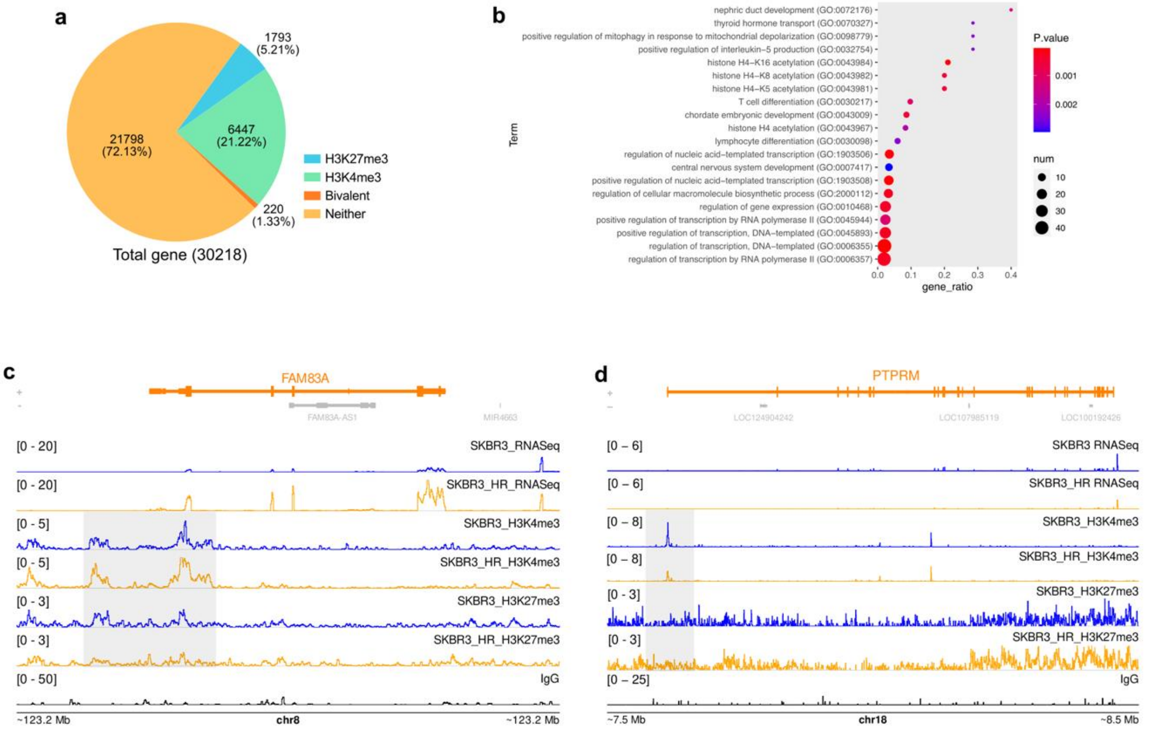

Supplementary figure 4

a, Promoter modification types of total genes in SKBR3 cells. **b,** GO biological processes enrichment of genes with bivalent promoters in SKBR3 cells. **c and d,** RNA-seq and CUT&Tag tracks at two gene locus (FAM83A and PTPRM) in SKBR3_HR and SKBR3 cells. Grey areas indicate the altered H3K27me3 or H3K4me3 peaks at bivalent promoter regions.

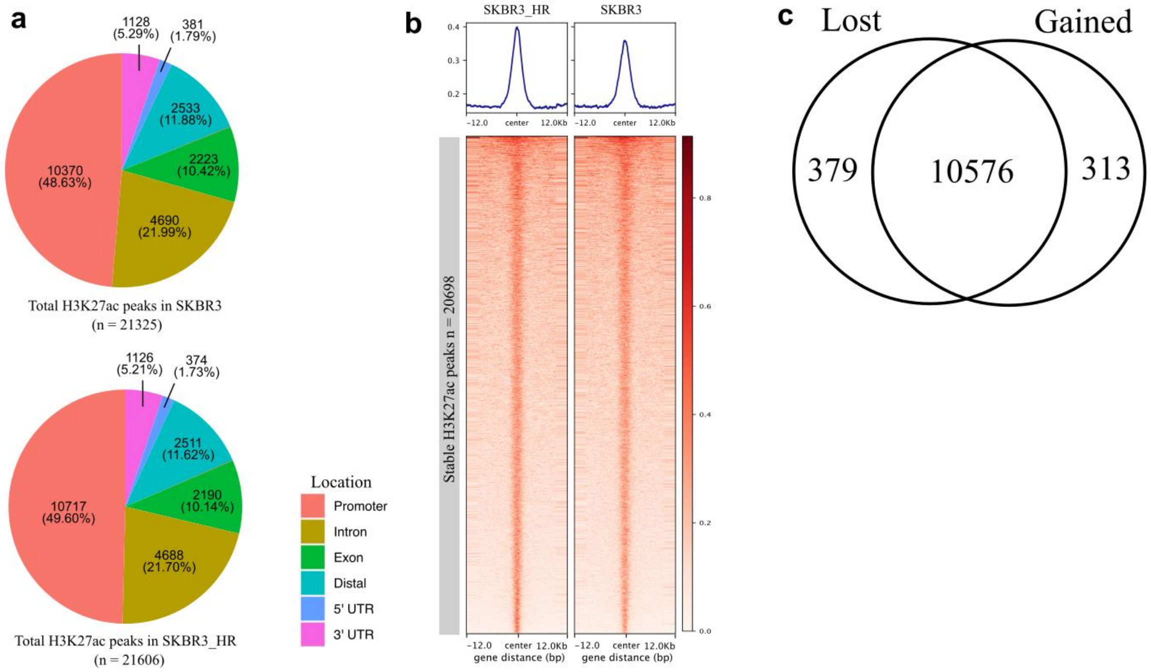

Supplementary figure 5

a, Distributions of total H3K27ac peaks in SKBR3 and SKBR3_HR cells. **b,** Stable H3K27ac peaks during secondary trastuzumab-resistance formation. **c,** Lost and gained active enhancers during secondary trastuzumab-resistance formation.

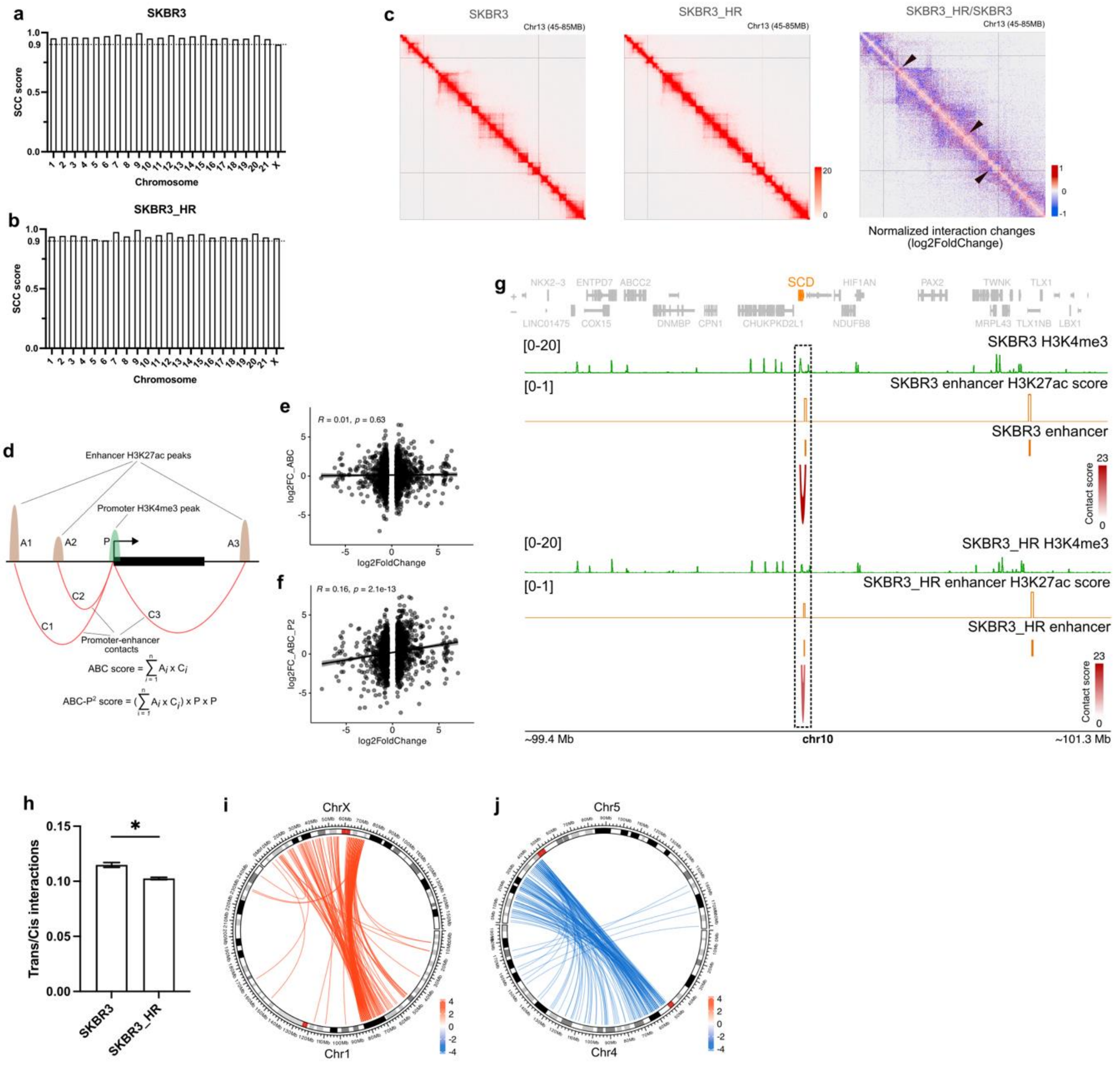

Supplementary figure 6

a and b, SCC correlation coefficients of different chromosomes between two replicates of SKBR3 and SKBR3_HR cells. **c,** Detailed intra-chromosomal interactions in certain chromosomal region (chr13: 45-85 MB) of SKBR3_HR and SKBR3 cells. Arrows indicate interaction changes. **d,** ABC model indicates the expression of gene is based on enhancer activity and promoter – enhancer contacts, while ABC-P^2 additionally considers the strength of promoter activity. **e and f,** Pearson

correlation coefficients between the log2 fold changes of ABC or ABC-P^2 scores and expression of significantly altered genes. **g,** Track plot showing the distributions of H3K4me3 peaks and enhances in ±1MB regions around SCD gene. The curve below links PEGES promoter and corresponding enhances and indicates the ABC scores between them according to the color. The dashed box indicates the promoter regions of PTGES in SKBR3 and SKBR3_HR cells. **h,** Ratio between inter- and intra-chromosomal interactions in SKBR3 and SKBR3_HR cells. **i,** Chord diagrams showing the top 0.5% most significant increased inter-chromosomal interactions between chromosome 1 and chromosome X. **j,** Chord diagrams showing the top 0.5% most significant decreased inter-chromosomal interactions between chromosome 4 and chromosome 5.

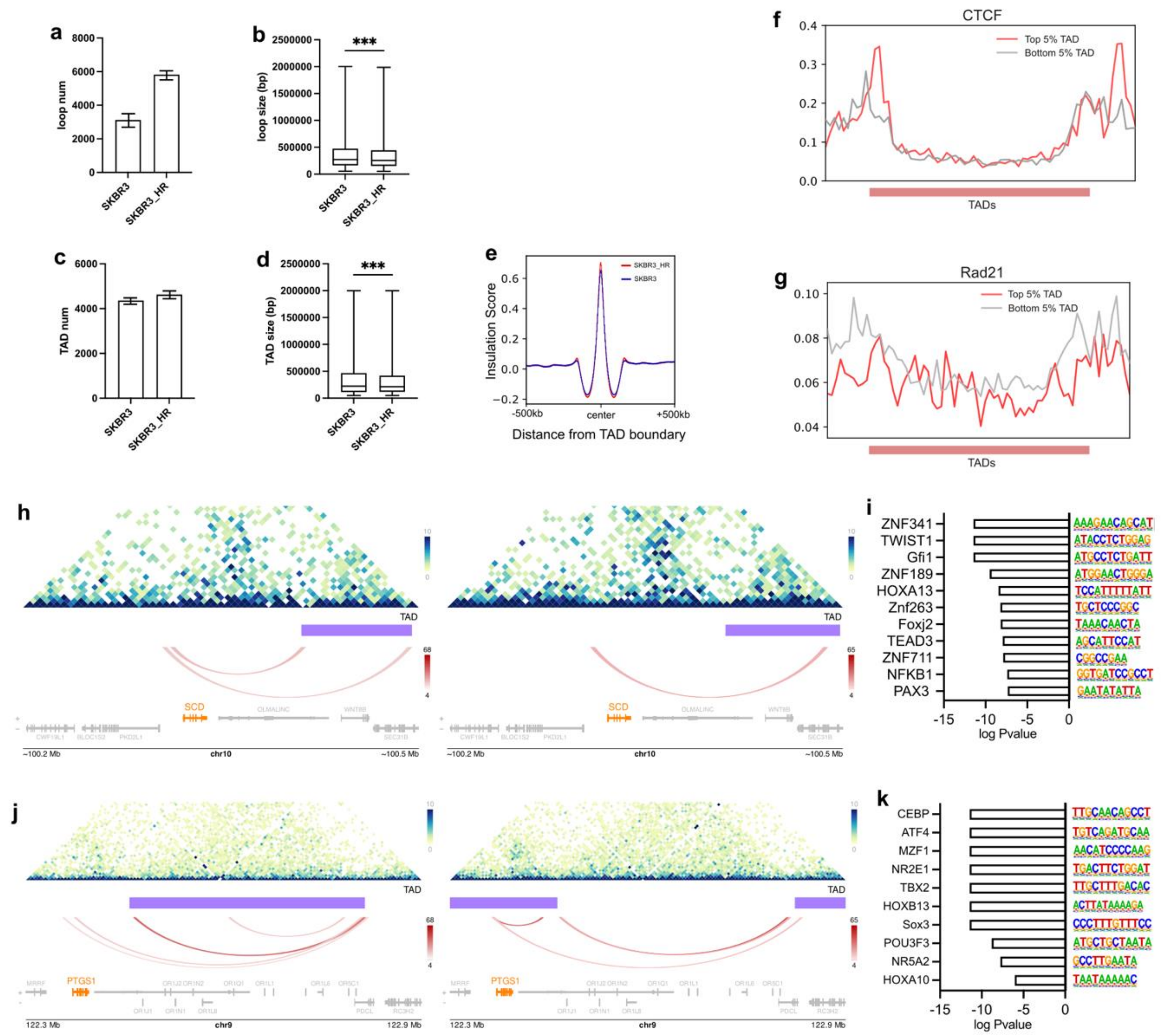

Supplementary figure 7

a, Total number of loops in SKBR3 and SKBR3_HR cells. **b,** Distributions of loop size in SKBR3 and SKBR3_HR cells. **c,** Total numbers of TADs in SKBR3 and SKBR3_HR cells. **d,** Distributions of TAD size in SKBR3 and SKBR3_HR cells. **e,** Insulation scores of all TAD boundaries in SKBR3 and SKBR3_HR cells. **f and g,** Distributions of CTCF and cohesin component (Rad21) peaks in TADs and TAD boundaries. The red line and grey line represent the top and bottom 5% altered TADs

during secondary trastuzumab formation. **h and i,** Track plots showing the locations of TADs and loops around different gene regions (SCD and PTGS1) in SKBR3 and SKBR3_HR cells.

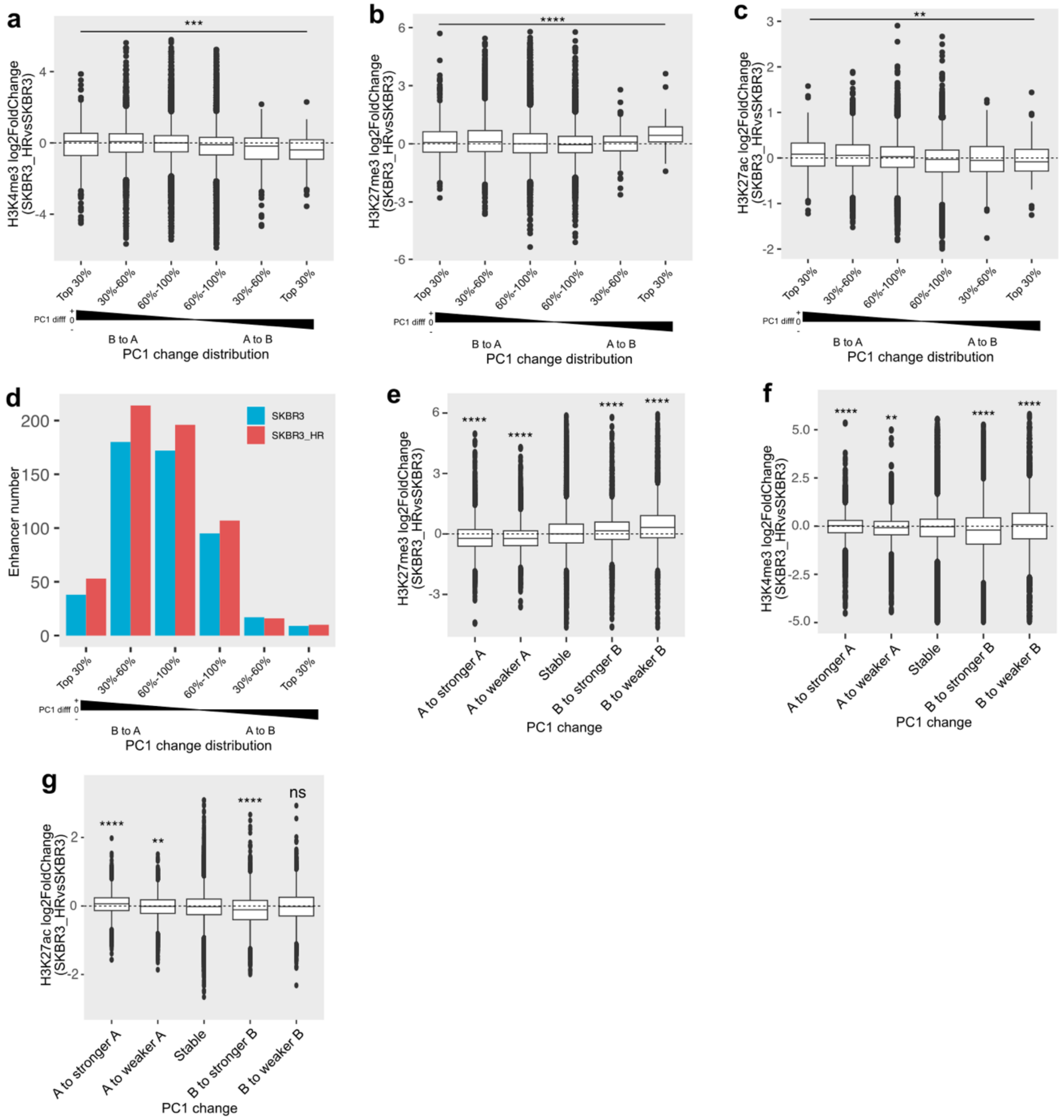

Supplementary figure 8

a-c, Distributions of log2 histone modifications changes (H3K27me3, H3K4me3 and H3K27ac) in different types of compartment changes (from top B to A partition to top A to B pattern). **d,** Distributions of enhancer in different types of compartment changes (from top B to A partition to top A to B partition). **e-g,** Distributions of log2 histone modifications changes (H3K27me3,

H3K4me3 and H3K27ac) in different types of compartment changes (A to stronger A, A to weaker A , stable, B to stronger B, B to weaker B).
